# Pacing-induced Cardiomyopathy

**DOI:** 10.5811/cpcem.2017.6.34398

**Published:** 2017-10-18

**Authors:** Alex Koo, Anna Stein, Ryan Walsh

**Affiliations:** * Madigan Army Medical Center, Department of Emergency Medicine, Joint Base Lewis-McChord, Washington; † Madigan Army Medical Center, Department of Internal Medicine, Joint Base Lewis-McChord, Washington

## Abstract

We present a case of pacing-induced cardiomyopathy. The patient presented with clinical symptoms of dyspnea, leg swelling, and orthopnea several months after a dual-chambered pacemaker was placed for third-degree heart block. The echocardiogram demonstrated a depressed ejection fraction. Coronary angiography was performed, which showed widely patent vessels. Single- and dual-chambered pacemakers create ventricular dyssynchrony, which in turn can cause structural, molecular changes leading to cardiomyopathy. With early intervention of biventricular pacemaker replacement, these changes can be reversible; thus, a timely diagnosis and awareness is warranted.

## INTRODUCTION

As the population of the United States continues to age, the number of implanted cardiac pacemakers has risen. Between 1993 and 2009, 2.9 million permanent pacemakers (PPM) were implanted in patients in the U.S., an increase in the use of these devices by nearly 56% within this time frame.^1^

Despite the improved quality of life pacemakers may provide for patients, these devices inherently have complications. The overall incidence of such adverse events is relatively low; however, complications of PPMs do exist with infection, lead malfunction, and venous thrombosis the most common.^2^ A more rare complication of PPM placement is pacing-induced cardiomyopathy. We present the first case of pacing-induced cardiomyopathy in the emergency medicine (EM) literature.

## CASE REPORT

A 70-year-old female with a medical history of coronary artery disease (CAD), type II diabetes, and three-vessel coronary artery bypass grafting (CABG) presented to the emergency department (ED) for dyspnea and productive cough. The patient endorsed increasing dyspnea on exertion, leg swelling, orthopnea, paroxysmal nocturnal dyspnea, and episodes of angina-equivalent chest pressure over the preceding two weeks. A chart review revealed the patient had been hospitalized four months prior for complete heart block with subsequent dual-chambered pacemaker placement. During the prior hospitalization, the patient’s echocardiogram demonstrated an ejection fraction of 55%.

On physical examination, the patient had crackles in both lung bases, jugular venous distention, and bilateral pitting lower-extremity edema. Her electrocardiogram (ECG) demonstrated a ventricular-paced, regular wide complex rhythm (Image). The N-terminal pro-brain natriuretic peptide (NT-pro-BNP) was elevated to >35,000 pg/mL and the troponin was elevated to 0.43 ng/mL. Cardiology was consulted for concern for a non ST-elevation myocardial infarction (NSTEMI) and acute heart failure.

During the hospital course, the patient had stable serial troponins, but a new echocardiogram demonstrated a decreased ejection fraction of 35%. The patient underwent left heart catheterization to evaluate for ischemic causes of cardiomyopathy and was found to have widely patent grafts and vessels without culprit lesions. The patient was medically optimized and routinely referred for biventricular (BiV) pacemaker placement after being diagnosed with pacing-induced cardiomyopathy. The patient returned to the ED two months later with exacerbation of her congestive heart failure symptoms, at which time she was upgraded to a BiV pacemaker.

## DISCUSSION

Pacing-induced cardiomyopathy (PICM) is a complication of single- and dual-chambered pacemakers, but is not well described in the EM literature. This complication is present in up to 9% of patients and is most prevalent within the first year after implantation.^3^ As such, it is a complication to consider in the ED with regard to a recent pacemaker implantation.

PICM is defined as a reduction in left ventricular ejection fraction (LVEF) of >10% after pacemaker placement. Additionally, paced beats must comprise >20% of the total QRS complexes.^4^ It is necessary to exclude other causes of a decreased LVEF including acute ischemia, valvular disease, and atrial arrhythmias before diagnosing PICM. Khurshid et al.^4^,^5^ retrospectively studied 1,750 patients with pacemakers to determine predictors of PICM. Male sex was an independent predictor of PICM. Additionally, wider paced QRS durations were associated with an increased PICM with a paced QRS duration > 150 ms 95% sensitive for PICM.

Interestingly, only half of patients with PICM had clinical evidence of heart failure as evidenced by the Framingham Heart Study: unexplained weight gain, dyspnea on exertion, paroxysmal nocturnal dyspnea, elevated jugular venous pressure, auscultatory crackles, S3 gallop, ascites, lower extremity edema, radiographic pulmonary edema or pleural effusion and need for initiation or uptitration of diuretic therapy.^5^

Our patient’s native rhythm was a right bundle branch pattern. During her hospitalization, she was monitored on telemetry with >90% paced beats, and paced QRS complexes ~188 ms. The combination of frequent pacing and widened paced QRS complexes contributed to an increased risk of our patient developing PICM. Shukla et al. also demonstrated wider paced QRS duration correlated with an increased incidence of hospitalizations for heart failure.^6^

**CPC-EM Capsule**

What do we already know about this clinical entity?

Pacing-induced cardiomyopathy is a complication of single- and dual-chamber pacemakers. New onset of heart failure within a year of placement is a common presentation.

What makes this presentation of disease reportable?

Pacemakers are commonplace and this complication with recent placement can be reversed with early recognition and appropriate referral.

What is the major learning point?

Pacing-induced cardiomyopathy should be included in the differential for a patient with new-onset heart failure with recent pacemaker placement.

How might this improve emergency medicine practice?

Understanding pacing-induced cardiomyopathy can help emergency physicians recognize and discuss this complication with our cardiology colleagues.

The pathophysiology of PICM is that right ventricular apical pacing changes the ventricular activation sequence, generating regions of early and delayed contraction causing important molecular changes. These result in a 20% decrease in systolic function, an increase in end-systolic volume and wall stress, and a delayed relaxation process. While the ventricular dyssynchrony is reversible with a BiV pacemaker, the changes become more difficult to reverse with time. BiV stimulation can clinically increase exercise tolerance and reduce heart failure hospitalizations.^7^

## CONCLUSION

To the best of our knowledge, this is the first case of pacer-induced cardiomyopathy in the EM literature. It is always necessary to exclude ischemia as a cause of new-onset heart failure; however, with pacemakers becoming commonplace, it is important for emergency physicians to be familiar with this life-threatening diagnosis that does not always present with the classic signs and symptoms of heart failure.

## Figures and Tables

**Image:**
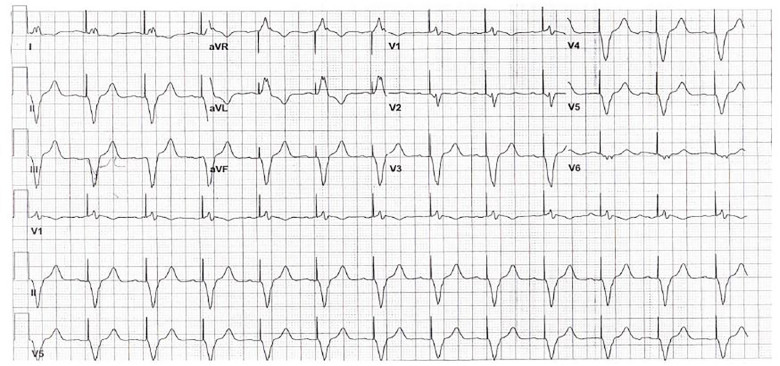
Paced electrocardiogram of patient with pacing-induced cardiomyopathy.
